# Supplementary material for: A Probiotic Bacillus amyloliquefaciens D-1 Strain Is Responsible for Zearalenone Detoxifying in Coix Semen
Source: Toxins (Basel). 2023 Nov 28;15(12):674. doi: 10.3390/toxins15120674 (PMC10747864; doi:10.3390/toxins15120674)
Supplement: Supplementary file 1 [file toxins-15-00674-s001.zip › toxins-2684051-supplementary.pdf]

A probiotic *Bacillus amyloliquefaciens* D-1 strain is responsible for zearalenone detoxifying in coix semen

Tao Deng [1,†], Yefei Chen [1,†], Jinqiang Zhang [1], Yanping Gao [1], Changgui Yang [1], Weike Jiang [1], Xiaohong Ou [1], Yanhong Wang [1], Lanping Guo [2], Tao Zhou [1,*] and Qing-Song Yuan [1,2,*]

[1] Resource Institute for Chinese & Ethnic Materia Medica, Guizhou University of Traditional Chinese Medicine, Guiyang 550025, China; td18300850597@163.com (T.D.); jqz18215598296@139.com (J.Z.); gaoyanping1121@foxmail.com (Y.G.); cgy15285135045@163.com (C.Y.); jwk_88@163.com (W.J.); yanhong824@hotmail.com (Y.W.)

[2] National Resource Center for Chinese Meteria Medica, State Key Laboratory for Quality Ensurance and Sustainable Use of Dao-di Herbs, Beijing 100700, China; glp01@126.com

* Correspondence: zhoutao700@gyctcm.edu (T.Z.); yuanqingsong006@gzy.edu.cn (Q.-S.Y.)

† These authors contributed equally to this work.

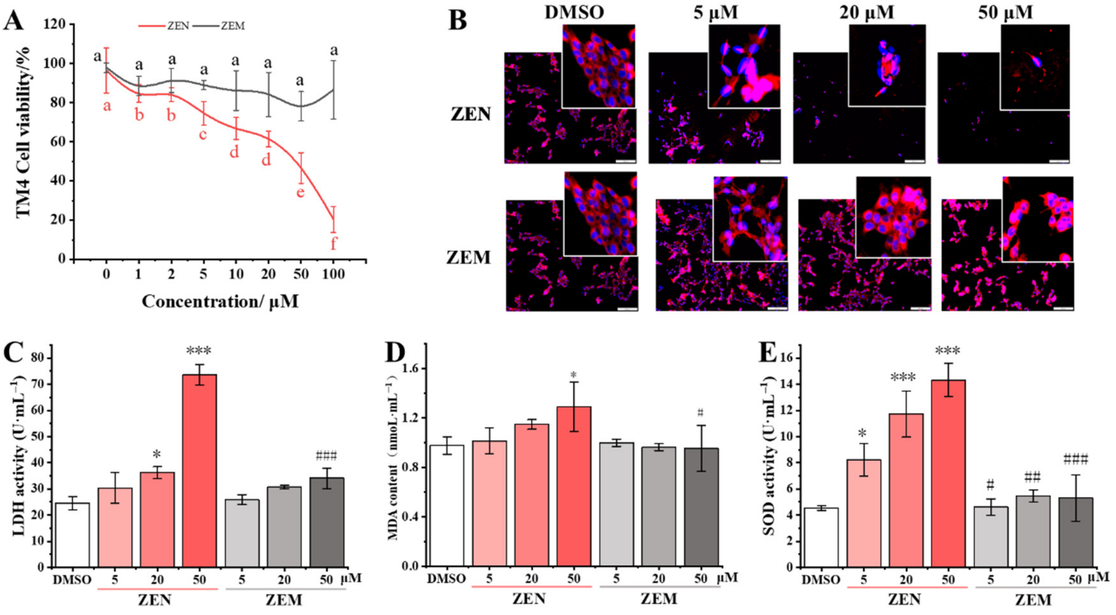

Figure S1. Effects of ZEN and its metabolite ZEM on TM4 cells. (**A**) Effects of ZEN and ZEM at different concentrations on the activity of TM4 cells. Letters a, b, c, d, e and f indicate that the ZEM group has a significant difference compared with the ZEN group at $p < 0.05$ levels with the same concentration (n = 6). (**B**) Immunofluorescence staining of TM4 cells. The cell membrane was stained red with Dil and the nucleus was stained blue with Hoechse 33342. (**C, D, and E**) Analysis of the release of LDH, the contents of MDA, and the activity of SOD in TM4 cells, respectively. *, **, and *** mean that the ZEN group and ZEM group have significant differences compared with the DMSO group at $P < 0.05$, $P < 0.01$, and $P < 0.005$ levels, respectively (n = 6). #, ##, and ### indicate that the ZEM group has a significant difference compared with the ZEN group at $P < 0.05$, $P < 0.01$, and $P < 0.005$ levels with the same concentration, respectively (n = 6). .

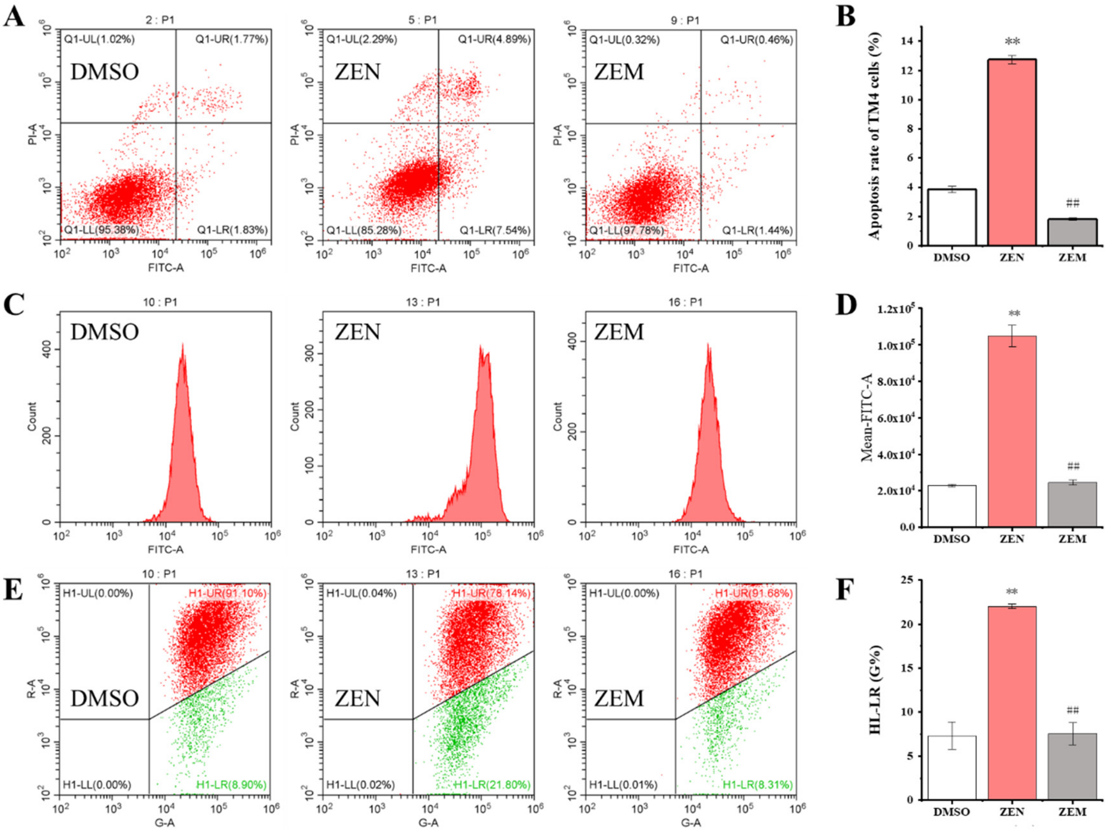

Figure S2. Effects of ZEN and its metabolite ZEM on the apoptosis (**A, B**), ROS (**C, D**), and mitochondrial membrane potential (**E, F**) of TM4 cells by flow cytometry. The Q1-UL region represents dead cells, the Q1-LR region represents early apoptotic cells, the Q1-LL region represents surviving cells, and the Q1-UR region represents late apoptotic cells. Normal mitochondria fluorescein red, and decreased or lost mitochondrial membrane potential fluorescein green. ** indicates that the ZEN group and ZEM group have significant differences compared with the DMSO group at $P < 0.01$ level (n = 6). ## indicates that the ZEM group has a significant difference compared with the ZEN group at $P < 0.01$ level (n = 6).

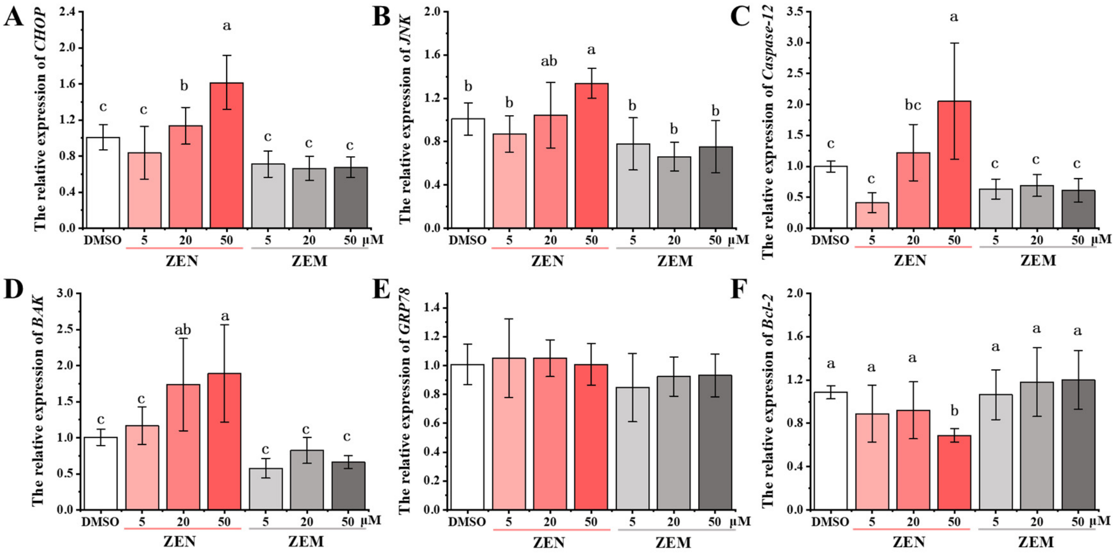

Figure S3. Effects of ZEN and its metabolite ZEM on the expression of apoptosis-related genes in TM4 cells by qRT-PCR. Different lowercase letters indicated significant differences among different treatment groups ($P < 0.05$) (n = 6).

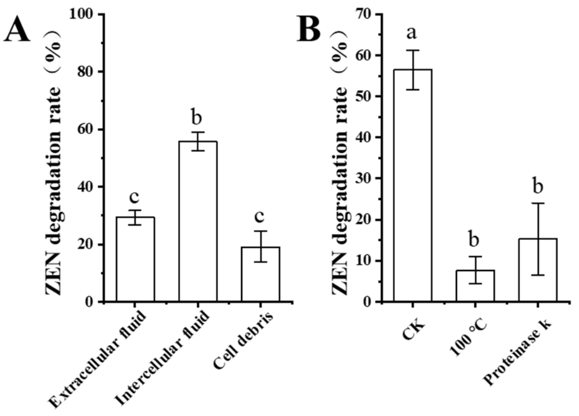

Figure S4. Degradation rate of ZEN by different components of D-1 strain. (**A**) The removal ability of ZEN in intracellular fluid, extracellular fluid, and cell debris of D-1 strain. (**B**) The ZEN degradation rate of intracellular fluid treated with protease K and boiling water bath. Different lowercase letters indicated significant differences among different treatment groups ($P < 0.05$) (n = 3).

Table S1. Primer's sequences of identification of strain D-1 and qRT-PCR.

Genes	Forward prime (5'-3')	Reward prime (5'-3')	Cell/ Strain
16S	CTACGGGMSGCAGCAG	GGACTACHVG GGTWTCTAAT	D-1
β-actin	CTGTCCCTGTATGCCTCTG	TTGATGTCACGCACGATT	
JNK	TCCTCCAAATCCATTACCTCC	CTCCAGCACCCATACATCAAC	
CHOP	TTCTCCTTCATGCGTTGCTTC	AAAACCTTCACTACTCTTGACCCTG	
Caspase-12	CTCAATAGTGGGCATCTGGGT	GAAGGTAGGCAAGACTGGTTC	HepG2
Bax	GCAAAGTAGAAGAGGGCAACC	ACTGGACAGCAATATGGAGCT	
Bcl-2	CTCAGGCTGGAAGGAGAAGAT	AAGCTGTCACAGAGGGGCTAC	
GAPDH	GAAGGTGAAGGTCGGAGTC	GAAGATGGTGATGGGATTTC	
P53	AATGCTCAGGGCAAGGAA	CAGGCAGAAGACTAACCAAG	
c-JNK	TCCTCCAAATCCATTACCTCC	CTCCAGCACCCATACATCAAC	
Caspase-8	GGATGGCCACTGTGAATAACTG	TCGAGGACATCGCTCTCTCA	TM4
Bax	CCCGAGAGGTCTTTTTCCGAG	CCAGCCCATGATGGTTCTGAT	
Bcl-2	GAGCCATCTCAGTGTGTGGAG	TGGGCAAACAGGTCAGCAG	
Fas	CTTTTCGTGAGCTCGTCTCTGA	CTCCCCAGAAGCGTCTTTGA	
